# Available Phosphorus in Forest Soil Increases with Soil Nitrogen but Not Total Phosphorus: Evidence from Subtropical Forests and a Pot Experiment

**DOI:** 10.1371/journal.pone.0088070

**Published:** 2014-02-04

**Authors:** Xingzhao Liu, Wei Meng, Guohua Liang, Kun Li, Weiqiang Xu, Liujing Huang, Junhua Yan

**Affiliations:** 1 South China Botanical Garden, Chinese Academy of Sciences, Guangzhou, China; 2 Paulownia Research and Development Centre, China Forestry Administration, Zhengzhou, China; 3 State Key Laboratory of Conservation and Utilization of Subtropical Agro-Bioresources, South China Agricultural University, Guangzhou, China; 4 Guangdong Provincial Academy of Environmental Science, Guangzhou, China; Dowling College, United States of America

## Abstract

This paper aims to establish evidence for available phosphorous (AP) binding with total nitrogen (N) in subtropical forest soils. Soil organic carbon (SOC), total N, total phosphorous (P) and AP concentration were measured for three contrasting forest types in southern China: Masson pine forest (MPF), coniferous and broadleaved mixed forest (CBMF) and monsoon evergreen broadleaved forest (MEBF). A pot experiment with N addition was conducted to confirm the dominant factor to affect on soil AP concentration. The results showed that mean soil total N concentration in 0–10 cm soil layer was 440±50 for MPF, 840±80 for CBMF and 1020±50 mg kg^−1^ for MEBF, respectively. The mean soil AP concentration in 0–10 cm soil layer was 2.67±0.87 for MPF, 2.65±0.58 for CBMF, 4.10±0.29 mg kg^−1^ for MEBF, respectively. The soil total N concentration could explain about 70% of the variations in soil AP concentration in the top 20 cm soil layers in the three forest types. A pot experiment with N addition also showed an increase of AP concentration from 2.56 to 5.63 mg kg^−1^, when N addition increased from 5 g to 17 g NH_4_NO_3_. Our results therefore suggested that N addition significantly increased soil AP concentration, which might be beneficial for stabilizing the net primary production of subtropical forests that were limited by soil AP. This finding may provide a theory basis for tropical and subtropical forests management.

## Introduction

Soil nitrogen (N) and phosphorus (P) are the most common macronutrients limiting plant growth under natural conditions. Inventories of global soil P found that soil P amount is lowest in the tropical and subtropical regions [1], [2] that account for about 40% of the global gross primary production and net carbon uptake over the past two decades [3], [4]. It is generally accepted that the total soil P gradually decreases as the result of weathering [5], ecosystems may decline at their advanced stage [6], which resulting in a decrease of biomass and diversity due to soil P limitation [7], [8]. Previous studies also found that available soil P reduced the responses of tropical forests to increasing CO_2_ concentrations [9], [10]. Under global increasing atmospheric CO_2_ and N deposition in the future, P limitation of net primary production (NPP) in terrestrial ecosystems, particularly tropical and subtropical forests, will likely exacerbate. However, a number of continuous eddy covariance measurements and long-term biomass inventories showed that old-growth forests can be significant carbon sinks, including tropical and subtropical forests [11]–[15].

The Dinghushan Biosphere Reserve (DBR) is located in the southern China experiencing a typical subtropical monsoon climate. The monsoon evergreen broadleaved forest (MEBF) is more than 400 years old [16], and is dominated by *Castanopsis chinensis*, *Schima superba*, *Cryptocarya chinensis*, *C. concinna*, and *Machilus chinensis*, which are typical species of forests at the advanced stage of succession in the subtropical area [17]. The prevailing theory of ecosystem biogeochemistry suggests that soil P availability would decrease in the advanced forest. A recent study also showed that soil P was relatively low in this region and P was possibly one of the limiting factors to tree growth in MEBF at the DBR [18]. However, long-term forest inventories and eddy covariance measurements showed that the NPP in MEBF was quite similar to that in the pioneer forest (Masson pine forest, MPF), or transition forest (coniferous and broadleaved mixed forest, CBMF) [19], [20].The reasons of such high ecosystem production in the P-limited, late successional subtropical forest are still unclear.

Remarkably, the advanced forest, MEBF, was saturated with N [21], as a result of very high input from atmospheric deposition (>30 kg N ha^−1^ yr^−1^) for much of the last two decades [22]. Accumulation of soil N can also affect soil P availability by changing soil ion balance and soil phosphatase production. Fertilization experiments demonstrated that N addition increased P cycle rate [23], [24]. Previous studies also suggested that N addition (mainly N deposition and N fertilizer applications) can significantly increase the availability of soil N and reduce P limitation in forest ecosystems [10], [25]. Some of those studies focused on the effects and mechanisms of soil N or P alone or N-P together, but very few studies examined the mechanisms of interaction between P limitation and N cycle, or whether N addition will alter P availability [10]. In tropical and subtropical regions where productivity is considered to be strongly P limited, a large carbon uptake above forest canopy [14] and accumulated in forest soil [16] have been found, but the mechanism of those observed large carbon uptake has not been understood yet. In this study, we hypothesize that N addition will increase available soil P production and reduce the affect of P limitation.

## Materials and Methods

### Ethics statement

The study site is maintained by South China Botanical Garden, Chinese Academy of Sciences. This site is within Dinghushan Forest Ecosystem Research Station. All necessary permits were obtained for the field study. The field study did not involve endangered or protected species. Data will be made available upon request.

### Site description

Our study site is located at the DBR (112.10° E and 23.10° N) in southern China. The reserve was established in 1950s to protect the natural forests, with an area of 1 133 ha and an elevation ranging from 10 to 1000 m above sea level. The local climate is subtropical monsoon climate, with a distinctive wet season (April-September) and dry season (October-March) within a year. The annual average rainfall is 1 700 mm, of which more than 80% falls in wet seasons. The mean annual temperature is 21.0°C, with average temperatures of 12.6°C and 28.0°C in the coolest month (January) and the hottest month (July) respectively. The mean annual relative humidity is 82%. Bedrocks are classified as Devonian sandstone and shale [26]. The lateritic red-earth at elevations below 400–500 m and yellow earth at higher elevations are major soil types in this region [27].

In the subtropical region, stand composition generally changes from coniferous to coniferous and broad-leaved mixed to broadleaved in the process of forest succession [17]. During natural succession, heliophytes (*e.g. S. superba* and *C. chinensis*) gradually invade coniferous forest to form mixed forest, and mesophytes (*e.g. C. concinna*, *C. chinensis*) subsequently invade mixed forest and eventually transform them into broadleaved forest. MPF, CBMF and MEBF mentioned above represent the forests in pioneer, transition and advanced stage, respectively [17]. Characteristics of these three forests are listed in Table 1.

**Table 1:** Stand characteristics of three forests in DBR.

	MPF	CBMF	MEBF	References
Successional stage	Pioneer	Transition	Advanced	[17]
Leaf area index	4.3	6.5	7.8	[28]
Biomass (kg C m^−2^)	6.13	8.21	14.52	[29]
Litter input (kg C m^−2^ year^−1^)	0.18	0.43	0.42	[30]
Soil bulk density (g cm^−3^)	1.49	1.22	1.09	[31]
Soil pH	4.02	3.92	3.80	[31]
Microbial biomass (10^6^ g^−1^ dry soil)	1.2	1.4	2.1	[32]
Fine root biomass in top soil (Mg C ha^−1^)	1.9	2.8	4.9	[31]

### Natural forest soil sampling

To quantify how soil nutrient varies with forest succession in DBR, soil samples were collected at depths of 0–10, 10–20, 20–40 and 40–60 cm using a 30 mm diameter auger, after removing organic layer of 1–2.5 cm above the soil surface. In each forest, three plots (10 m × 10 m) were established. In each plot, 3 randomly located subplots were selected. In each subplot, four augers of soil in different layers were taken and mixed to make up a composite sample, resulting in 9 composite samples for each soil layer for each forest type. All samplings were collected in September-October 2005.

### Pot-based N addition experiment

Seedlings of four species were selected for the pot experiment, among which *P. massoniana* is the pioneer species, while *S. superb*, *C. chinensis* are dominant native tree species. *Ormosia pinnata* was chosen as the main legume tree species to evaluate the influences of N-fixing on soil nutrient availability. Clay pots with 33 cm in diameter and 30 cm in depth were used for cultivating tree seedlings. The soil was collected from the transitional forest at DHR. About 18.6 kg mixed soil (fresh weight) was put into one pot. Seedlings of different species with similar height were collected from the forests. One seedling was planted into one pot.

To simulate the soil N and P conditions in subtropical forests of China, we employed seven levels of N addition (Control, 5, 8, 11, 13, 15, 17 g NH_4_NO_3_ for each time), four replicates for each level. For each pot, NH_4_NO_3_ was dissolved with 1 L spring water and then added to the pot soil, with exceptions in the control treatment where no NH_4_NO_3_ was added but only the equal volume of spring water. A total of 10 times of N addition were conducted during the experiment with a frequency of every 20 days.

**Figure 1:**
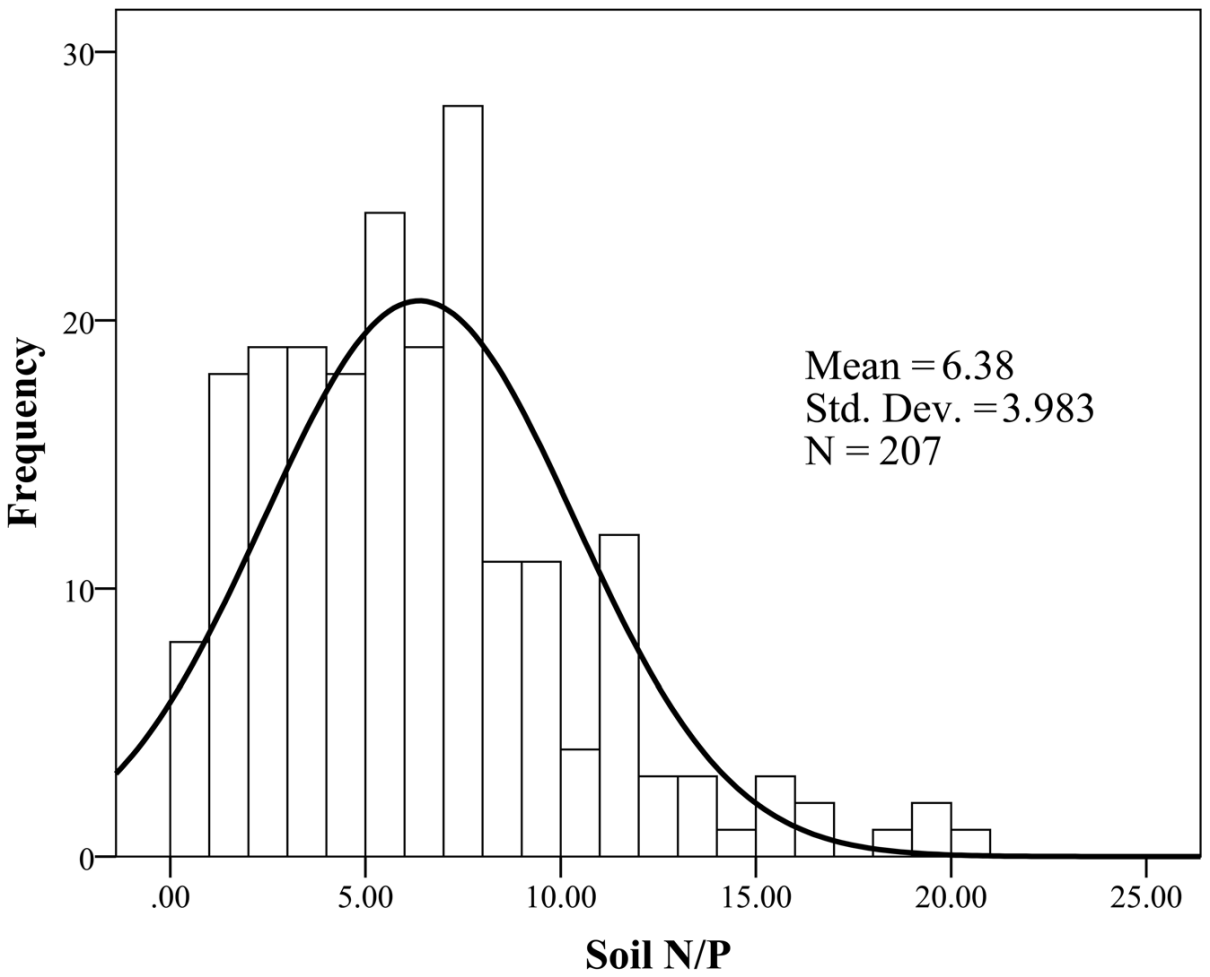
Frequency histograms of soil N/P ratio in six subtropical forests in China.

The amount of N addition was designed based on the range of soil N/P ratios of six subtropical forests in China (unpublished data, Fig. 1) and the background data of the pot soil in this experiment (Table 2). The background N/P ratio of pot soil was about 5. We considered that it is unnecessary to simulate soil N/P ratios less than 5 because soil in the DHR is rich in N but poor in P and this trend is continuing [33].

**Table 2:** The properties of pot soil before N addition treatment.

	SOC (mg kg^−1^)	N (mg kg^−1^)	P (mg kg^−1^)	AP (mg kg^−1^)	N/P
Mean	32230	1080	220	3.061	4.960
*n*	50	50	50	5	50
*s.d.*	3661	140	30	1.25	0.54

### Measurement

Soil was sampled with a soil auger (diameter 2.5 cm) from surface to bottom in each pot after the tenth N addition. The samples were dried by free air and sieved (2 mm) to remove plant residues, and mixed thoroughly by hand. To reduce the analytical error, the average value of two replicates per sample was used and all data were expressed on an oven dry weight basis. Soil organic carbon (SOC), total N, total P and available P (AP) concentration were measured by standard element analyzer. The determination was in accordance with standard methods for observation and analysis in Chinese Ecosystem Research Network. SOC concentration was determined by potassium dichromate oxidation [34]. Semi-micro Macro Kjeldahl method was adopted to analyze soil total N concentration [35]. Soil total P concentration was measured by acid dissolve molybdenum and antimony anti-spectrophotometry (Spectrum lab 24, Shanghai, China). Soil AP was extracted with ammonium fluoride (NH_4_F, 0.03 mol L^−1^) and hydrochloric acid (HCl, 0.025 mol L^−1^) [36], and measured by UV-Vis spectrophotometer (Spectrum lab 24, Shanghai, China).

### Data analysis

Data were tested for normality and equality of variance. All analyses were conducted with the SPSS statistical package for Windows (16.0, SPSS, Inc, Chicago, IL). Differences of SOC, total N, total P and AP concentration among the three forests were tested by One-Way ANOVA. The effects of treatments on pot soil total N, total P, AP concentration and other soil parameters were analyzed using one way ANOVA with N addition as the fixed factor. Correlations between SOC, total N, total P and AP concentration were analyzed using Pearson correlation tests.

## Results

### Patterns of SOC and soil total N in three successional forests

**Figure 2:**
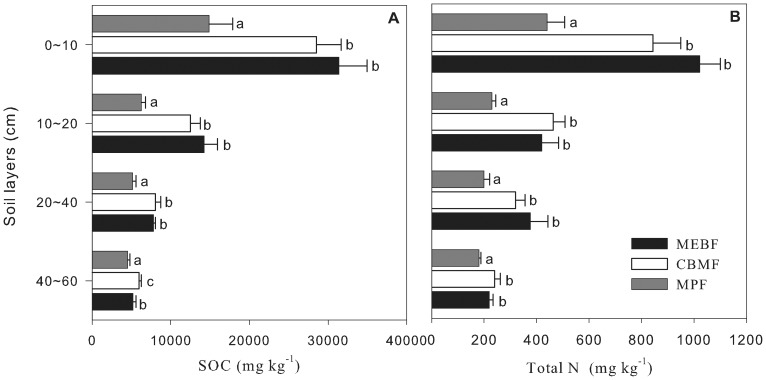
SOC and total N concentrations among three forest types at different succession stages in DBR. Error bars indicate one standard deviation. Different letters indicate significant differences at the confidence level of *P*<0.05 in the same soil layer among the three forests. (OneWay ANOVA, *P*<0.05).

Fig. 2A showed that SOC concentration in the three forests decreased with soil depth. SOC concentration in 0–10 cm soil layer was about 2–5 times higher than that in 40–60 cm soil layer for the three forests (Fig. 2A). Mean SOC concentration in 0–10 cm soil layer increased with the forest succession, being 14868±2987 for MPF, 28508±3175 for CBMF and 31406±3572 mg kg^−1^ for MEBF. This increase was significant between pioneer (MPF) and transitional (CBMF) forests (*P*<0.05), while no significant difference between transitional (CBMF) and advanced forests was found (*P*>0.05).

Fig. 2B showed that soil total N concentration in the three forests also decreased with soil depth. In each forest, soil total N concentration in 0–10 cm soil layer was significantly greater than that in each of the other three soil layers (*P*<0.05). There was no significant difference in soil total N concentration among soil layers of 10–20 cm, 20–40 cm and 40–60 cm (*P*>0.05). Mean soil total N concentration in 0–10 cm soil layer increased with the forest succession, being 440±50 for MPF, 840±80 for CBMF to 1020±50 mg kg^−1^ for MEBF, respectively. The difference in soil total N concentration between MPF and CBMF or MEBF was significant (*p*<0.05), and insignificant (*P*>0.05) between CBMF and MEBF.

### Patterns of soil total P and soil AP in three successional forests

**Figure 3:**
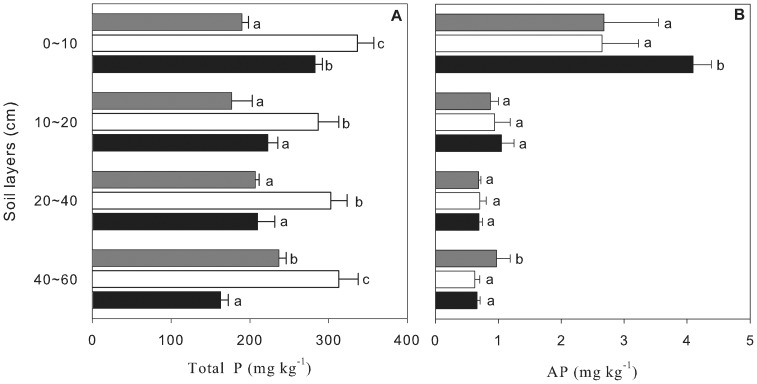
Soil total P and AP concentrations among three forest types at different succession stages in DBR. Error bars indicate one standard deviation. Different letters indicate significant differences at the confidence level of *P*<0.05 in the same soil layer among the three forests. (OneWay ANOVA, *P*<0.05).

Fig. 3A showed that the variations in soil total P concentration with soil depth were quite different among the three forests. In the pioneer forest, soil total P concentration increased with soil depth and this increase was only significant between 20–40 cm and 40–60 cm soil layers (*P*<0.05). In the transitional forest, soil total P concentration was not significant difference among the different soil layers (*P*>0.05). In the advanced forest, soil total P concentration decreased with soil depth and this decrease was only significant between 0–10 cm and 10–20 cm soil layers (*P*<0.05).

Soil total P concentration was significantly greater in the transitional forest when compared to the pioneer or advanced forest (*P*<0.05). Mean soil total P concentration from 0 to 60 cm was 209.2 for the pioneer, 309.3 for the transition and 208.7 mg kg^−1^ for the advanced forest, respectively. In the 0–10 cm soil layer, soil total P concentrations was 190±10 for MPF, 340±20 for CBMF and 280±10 mg kg^−1^ for MEBF, respectively (Fig. 3A).

However, soil AP concentration in 0–10 cm soil layer was the least in the transitional forest, and the highest in the advanced forest. The mean soil AP concentration in 0–10 cm soil layer was 2.67±0.87 for MPF, 2.65±0.58 for CBMF, 4.10±0.29 mg kg^−1^ for MEBF, respectively. In each of the three forests, soil AP concentration in 0–10 cm soil layer was significantly greater than that in each of the other soil layers (*P*<0.05). There was no significant difference in soil AP concentration among soil layers of 10–20 cm, 20–40 cm and 40–60 cm (*P*>0.05) (Fig. 3B).

### Correlations of soil AP with SOC, total N and total P

**Figure 4:**
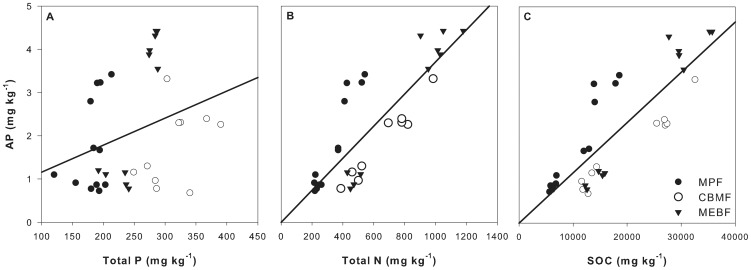
The correlation between soil total P (a), total N (b), SOC (c) and AP concentrations in 0–20 cm soil of three forests in DBR. (a): AP = 0.0062 P+0.536, *R^2^* = 0.096, *P* = 0.920; (b) AP = 0.003784 N-0.0003, *R^2^* = 0.699, *P*<0.0001; (c): AP = 0.0001 SOC-0.0016, *R^2^* = 0.713, *P*<0.0001.

As shown in Fig. 3, the patterns (distribution along soil depth and variation with forest succession) of soil AP concentration were quite different with soil total P concentration. Therefore, the variation in soil AP concentration was not consistent with the variation in soil total P concentration. As a test of this result, we examined the relation between soil total P concentration and soil AP concentration (Fig. 4A). A weak positive relationship (*R^2^* = 0.096) was found between soil total P concentration and soil AP concentration. However, the degree of scatter in the plotted points (*P* = 0.920) suggests that the trends were as expected.

However, the distribution of soil AP concentration along soil depth were quite similar to the distribution of soil total N concentration or SOC concentration in each of the three forests. We plotted soil AP concentration against soil total N or SOC concentration in the soil layers of 0–10 cm and 10–20 cm in the three forests together (Fig. 4B and 4C). A linear regression fitted well between soil AP concentration and soil total N or SOC concentration. Soil total N or SOC concentration could explain about 70% of variations in soil AP concentration.

### Effects of soil total N or SOC on soil AP concentration

**Figure 5:**
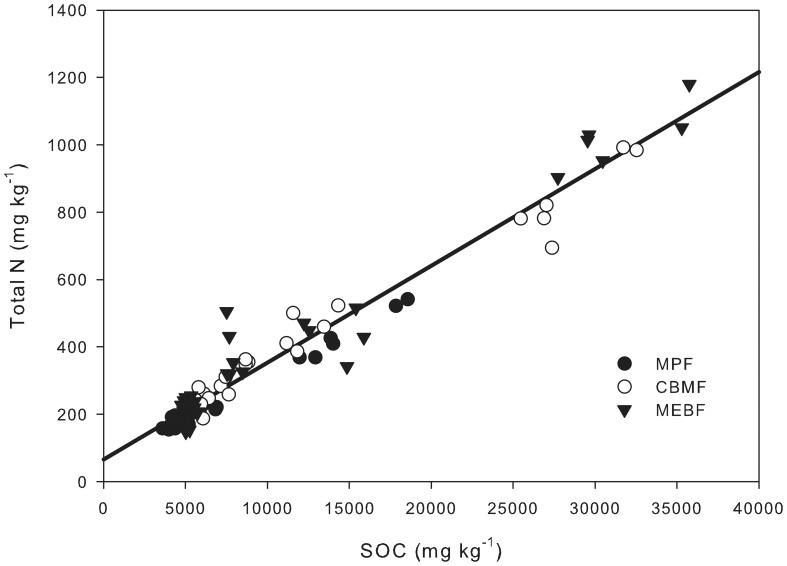
The correlation between soil total N and SOC concentrations in 0–20 cm soil of three forests in DBR. N = 0.029 SOC-66.627, *R^2^* = 0.954, *P*<0.0001.

**Figure 6:**
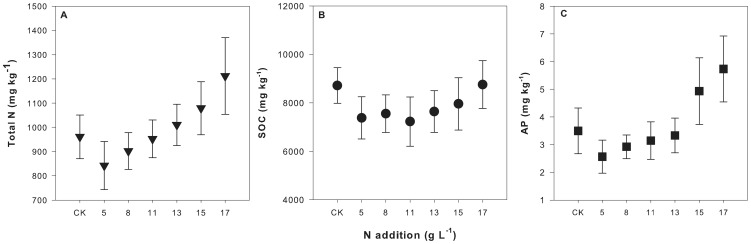
Variation of pot soil total N (A), SOC (B) and AP (C) concentrations with the rate of N addition. Error bar represents one standard deviation. (A): *r* ^2^ = 0.307, *P* = 0.000; (B): *r* ^2^ = 0.029, *P* = 0.072; (C): *r* ^2^ = 0.222, *P* = 0.000.

As mentioned above, each of soil total N and SOC could explain much of the variations in soil AP concentration. Both of soil total N and SOC increased with the forest succession and showed co-variation significantly (Fig. 5). Therefore, it was difficult to distinguish which was the dominant factor to drive the variations in soil AP concentration based on the field measurements. To confirm the dominant factor on soil AP concentration, a pot experiment with N addition was employed. The results from this experiment showed that soil total N concentration increased with N addition level (Fig. 6A). This increase was significant under the different N addition levels (*P*<0.05) (Fig. 6). SOC concentration also increased with N addition level (Fig. 6B), but this increase was not significant under the different N addition levels (*P*>0.05) (Fig. 6). The variations in soil AP concentration with N addition level were similar to soil N concentration (Fig. 6C). There was significant difference in soil AP concentration under different N addition levels (*P*<0.05) (Fig. 6).

**Figure 7:**
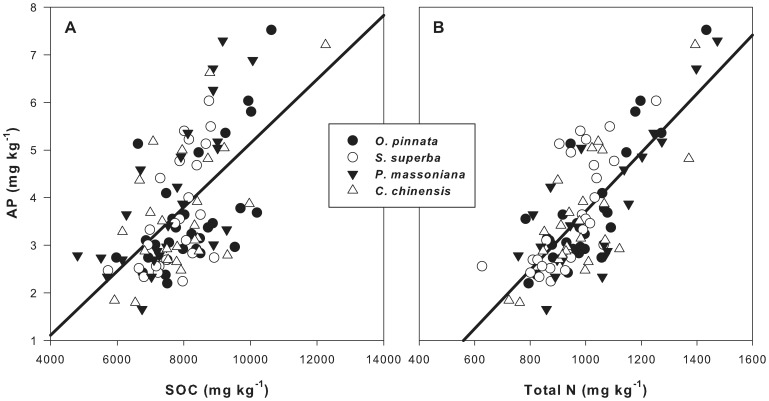
The correlation between pot soil C (A), N (B) and AP concentrations. (A): AP = 0.00067 SOC-1.577, *R* ^2^ = 0.359, *P*<0.0001; (B): AP = 0.006 N-2.443, *R* ^2^ = 0.578, *P*<0.0001

Soil total N concentration increased from 960.7 (control) to 1212.3 mg kg^−1^ (highest N addition level) (Fig. 5). Soil AP concentration increased from 3.5 (control) to 5.7 mg kg^−1^ (highest N addition level) (Fig. 5). We found a strong relationship (AP = 0.006 N-2.443, *R* ^2^ = 0.578, *P*<0.001) between soil AP concentration and total N concentration. Soil total N could explain about 60% of the variations in soil AP concentration (Fig. 7). However, the linear correlation of soil AP concentration with SOC was much weaker than that with soil total N concentration (AP = 0.0007 SOC-1.577, *R* ^2^ = 0.359, *P*<0.001). SOC only explained about 36% of variations in soil AP concentration (Fig. 7).

## Discussion

### Why can N addition increase AP in subtropical forest soil?

Soil AP mainly came from the cleavage of soil organic P via biochemical P mineralization or decomposition of soil organic matter via biological mineralization. Both plant roots and soil micro-organism produced phosphatase for biochemical P mineralization [37]. Secreted by plants or microbes, extracellular phosphatases hydrolyzed phosphodiester-bonded P to phosphate ions that were available for uptake by plant roots [37]. It was reported that phosphodiester-bonded P accounted for 20% to 80% of soil organic P [38].

Phosphatase came from the physiological activity of soil microorganisms. Meanwhile, phosphatase was a constitutively N-rich (15% N, [24]) class of enzymes. The addition of N could provide the necessary element for the production of phosphatase [23], [24]. The soil in MEBF in the DBR was relatively richer in N, therefore it produced more AP than other two forests.

### P limitation and C allocation in forests

N addition can accelerate soil P cycle by increasing the production of extracellular phosphatase enzyme. However, N was not the only factor which affects P cycle. The physiological activity of soil microorganisms, in which extracellular phosphatase enzyme was produced, depends on available C for growth and energy. Consequently, about 36–71% of variation of AP in the soil could be explained by SOC (see Fig. 4 and 7A). Therefore, C was another factor influencing AP in soil. The root exudates and dead root can be used by soil microbe to produce enzymes, which could increase soil AP [23], [39].

In turn, P limitation can impact C cycle. P limitation was very common in old-growth forests, especially at low latitudes [6]. In this case, plants should develop strategies for adapting to low P soil, and make necessary physiological adjustments to alleviate P limitation. The activation of insoluble phosphate by the interaction of root and soil microbe was an effective mechanism. However, this strategy required high C input. Plants allocated new biomass C preferentially to roots for the growth of root and exudates, both of which could increase the absorption of P. The allocation of C to belowground, to some extent, would decrease the aboveground biomass, even the total biomass if the proportion of exudate was too high. Actually, the exudates accounted for a significant proportion of annual net primary production. Some studies demonstrated that the annual rate of exudates production in rhizosphere can account for 15–23% of total net primary production [40], [41]. Marschner (1995) indicated that the amount of organic matter released from root to rhizosphere accounted for 1–30% of the photosynthetic carbon assimilation [42].

The changes of C allocation in P-limitation forests would bring a great amount of C into soil. MEBF in DBR, older than 400 years, was considered as a P-deficiency and N-adequacy ecosystem [43]. However, it has accumulated C with a steady rate during the last 23 years [20]. Furthermore, the standing biomass and annual litter decreased [20], [43] and soil C increased [16], [43], [44]. Carbon invested into belowground will contribute to the accumulation of soil C. A shift in C allocation toward belowground was the physiological adjustments to alleviate P limitation in MEBF. As a result, the AP content in MEBF was highest among the three forests in DBR. Our measurements showed that annual NPP in MEBF had been relatively stable for at least twenty years. Consequently, C allocation change was an effective adaptation strategy under P limitation in old-growth forests.

### The mechanism of P limitation in subtropical forest ecosystems

Available soil P changed along a forest successional gradient [2], [6]. As forest canopy developed, the microenvironment below the forest became wetter and shadier, which will increase chemical weathering of parent material, and therefore soil P lost [5]. As a result, P became limiting to plant growth in the old-growth forests [6], [33].

Because of input and output of P to soil depends on many other geological and ecological processes as well as plants themselves, the mechanisms of P limitation were variable. Vitousek *et al.* concluded six mechanisms that can cause P limitation to terrestrial ecosystems: P depletion, soil barriers, transactional, low-P parent materials, P sinks, and anthropogenic forcings [10]. These mechanisms were not exclusive. It was likely that P limitation in any ecosystem will be more or less controlled by more than one mechanism.

Perhaps the most likely cause of P limitation to old-growth tropical forest ecosystems was depletion-driven P limitation, the progressive loss of P that occurs during long term soil and ecosystem development [10].The regional bedrock in the DBR was thick sandstone and sand shale from Devonian [26] that has undergone a very long period of weathering. At the same time, locating in low-subtropical made their parent material P impossible to be rejuvenated or subsidized by glacial or periglacial processes. Except for the topsoil which was strongly affected by biology, depletion of P was evident in the deep soil (40–60 cm) along the forest succession in the DBR. Much of P may be lost via leaching of dissolved organic P [45].

The other possible mechanism of P limitation in the DBR was anthropogenic P limitation. The DBR locates in East Asia, where human-enhanced N deposition was one of highest in the world [46]. The annual N wet deposition amounted to 38.4 kg N ha^−1^·yr^−1^ in the DBR [22] and soil was saturated with N in the old-growth forest [21]. The pot fertilization experiments further demonstrated that N addition can accelerate soil P cycle by increasing the production of extracellular phosphatase enzyme [23], [24]. However, the increase in P cycling was insufficient to balance the increased rate of N inputs, hence, P often became limiting.

## Conclusions

The field surveys of forest soil total N, SOC, total P, AP concentration and the N-addition experiments in Dinghushan, Southern China, allowed us to estimate the relationship between soil total N, SOC, total P and AP. The main findings of the present study are:

1. Comparing to the two younger forests (MPF and CBMF), the advanced forest (MEBF) had greater AP concentration but less total P concentration in soil.
2. The concentration of soil total N or SOC increased with the forest succession and showed a similar pattern with soil AP concentration. A significant correlation was found between soil AP concentration and soil total N concentration (*R* ^2^ = 0.713, *P*<0.001) or SOC concentration (*R* ^2^ = 0.699, *P*<0.001) at top 20 cm soil layer. However, there was very weak correlation (*R* ^2^ = 0.096, *P* = 0.920) between soil P concentration and AP concentration.
3. Results from the pot experiment with N addition showed that soil AP concentration increased with N addition. Soil total N concentration could explain about 60% of the variations in soil AP concentration.
